# Water and Life: The Medium is the Message

**DOI:** 10.1007/s00239-020-09978-6

**Published:** 2021-01-11

**Authors:** Moran Frenkel-Pinter, Vahab Rajaei, Jennifer B. Glass, Nicholas V. Hud, Loren Dean Williams

**Affiliations:** 1 NASA Center for the Origins of Life, Atlanta, GA USA; 2 NSF-NASA Center of Chemical Evolution, Atlanta, GA USA; 3 School of Chemistry and Biochemistry, Georgia Institute of Technology, 315 Ferst Drive NW, Atlanta, GA 30332-0400 USA; 4 School of Earth and Atmospheric Science, Georgia Institute of Technology, 311 Ferst Drive NW, Atlanta, GA 30332-0340 USA

**Keywords:** Metabolism, Metabolite, Glycolysis, Amino acid, Translation, Oxidative phosphorylation

## Abstract

Water, the most abundant compound on the surface of the Earth and probably in the universe, is the medium of biology, but is much more than that. Water is the most frequent actor in the chemistry of metabolism. Our quantitation here reveals that water accounts for 99.4% of metabolites in *Escherichia coli* by molar concentration. Between a third and a half of known biochemical reactions involve consumption or production of water. We calculated the chemical flux of water and observed that in the life of a cell, a given water molecule frequently and repeatedly serves as a reaction substrate, intermediate, cofactor, and product. Our results show that as an *E. coli* cell replicates in the presence of molecular oxygen, an average in vivo water molecule is chemically transformed or is mechanistically involved in catalysis ~ 3.7 times. We conclude that, for biological water, there is no distinction between medium and chemical participant. Chemical transformations of water provide a basis for understanding not only extant biochemistry, but the origins of life. Because the chemistry of water dominates metabolism and also drives biological synthesis and degradation, it seems likely that metabolism co-evolved with biopolymers, which helps to reconcile polymer-first versus metabolism-first theories for the origins of life.

**Electronic supplementary material:**

The online version of this article (10.1007/s00239-020-09978-6) contains supplementary material, which is available to authorized users.

## Introduction

Water is the most abundant liquid on the surface of the Earth (Weingärtner et al. 2000) and life as we know it demands water (Brack 1993; Ball 2017). Biochemist Albert Szent-Györgyi stated: “Water is life’s matter and matrix, mother and medium. There is no life without water” (Szent-Györgyi 1971). Most cells are around 65% water by volume and 70% by mass (Milo and Phillips 2015), and living organisms have developed sophisticated mechanisms for appropriating and conserving water (Milo and Phillips 2015). The properties of water, and its roles as the biological medium, have been extensively characterized. Water drives folding and assembly of biopolymers (Kauzmann 1959; Radzicka et al. 1988; Sundaralingam and Sekharudu 1989; Barron et al. 1997) and supports the formation of membranes and other biological structures. Similarly, it is well documented that water is produced, consumed, altered, or utilized transiently during enzymatic reactions (Testa and Kraemer 2007; Nagano et al. 2015). Water is a metabolite (Kim et al. 2007), which is defined as an intermediate or end product of metabolic reactions (Venes 1940), even though it is usually not described as such (for example, see Fell and Wagner 2000; Testa and Kraemer 2007; Bennett et al. 2009; Moradi et al. 2017).

Water defines the chemistry of biology and is the dominant chemical actor in the connected chemical reactions that maintain the cellular living state, i.e., in metabolism. Water is chemically involved in a plurality of biochemical transformations. The ability to chemically consume and produce water is the unifying theme of organic and inorganic molecules of biology. Essentially all biological molecules, large and small, are products of or substrates for biochemical reactions that transform water.

A survey here suggests that one third to one half of known enzymatic reactions consume or produce water. This fraction under-represents involvement of water in enzymology because it omits reactions that use water transiently during catalysis. A second survey tallied the number of water molecules involved in two dominant cellular metabolic hubs: protein synthesis and oxidative phosphorylation. The results show that an average water molecule is chemically transformed or is mechanistically involved in catalysis about 3.7 times by these two processes as an *Escherichia coli* (*E. coli*) replicates in the presence of molecular oxygen (O_2_). Based on these quantitative data, we define water as the chemical cornerstone of biological processes and challenge the notion or even the possibility of an inert biological solvent.

Notably, our quantitative demonstration of water as the dominant reactive species in biology is distinct from previously defined “metabolic waters” (Mellanby 1942). That term focuses on water molecules produced by specific metabolic processes and excludes many other transformations of water in biochemical processes, such as water molecules involved (i) in hydrolysis, (ii) as intermediates, or (iii) mechanistically in enzymes.

## Results

### Reactive Core of Biology: Defining Transformations of Water

**Fig. 1:**
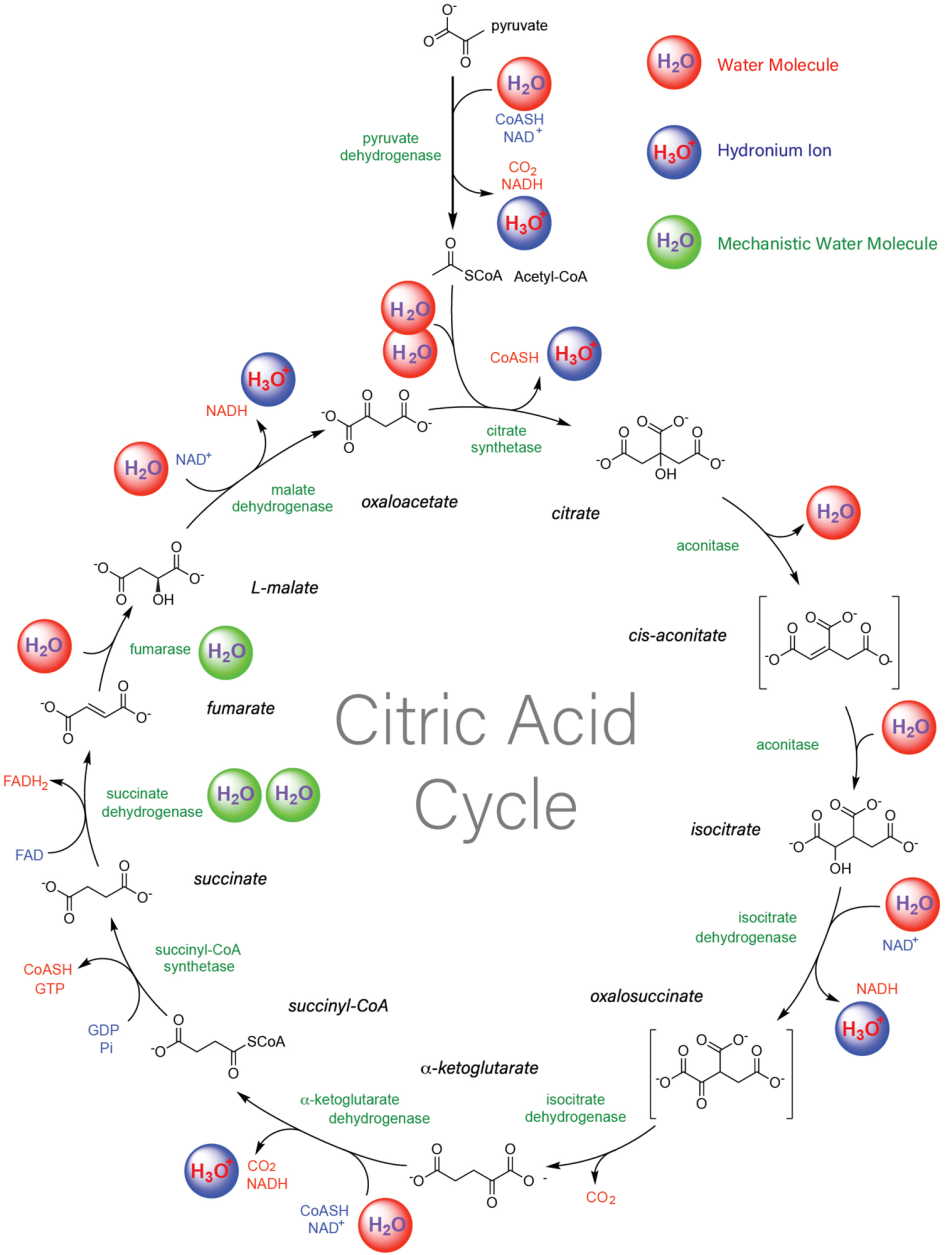
Chemical transformations of water by the citric acid cycle. Eight enzymes (green text) catalyze a series of reactions that in total, consume three water molecules, produce one water molecule, protonate three water molecules, and convert an acetyl group to two carbon dioxide molecules. The series of reactions produces three NADH, an FADH_2_, and a GTP. Unprotonated water molecules are indicated by red spheres. Protonated water molecules are blue. Water molecules that are mechanistically involved in the reactions are green (Color figure online)

Metabolism is a complex network of chemical reactions that are kinetically and thermodynamically linked by common substrates, intermediates, products, and effectors. Water is a key agent of cooperation of a core set of metabolic reactions. The production, consumption, and catalytic utilization of water in the citric acid cycle are illustrated (Fig. 1). Reactions that transform water include (i) condensation dehydration and intramolecular dehydration reactions, that chemically produce water; (ii) hydrolysis reactions, that chemically consume water; (iii) reactions that use water mechanistically during catalysis; (iv) reactions that produce protons, hydroxide, carbon dioxide, peroxide, etc., which cause downstream chemical reactions of water; (v) reactions that split water (e.g., in photosynthesis); and (vi) changes in metal coordination that release or absorb water*.*

Many water-consuming reactions are catalyzed by the hydrolase family of enzymes and involve cleavage of pyrophosphates, esters, peptides, acetyls, ethers, etc. (Supporting Fig. S1). Water is also consumed by many oxidoreductase, lyase, and transferase enzymatic reactions. Some water-consuming reactions are enabled by acid–base chemistry: chemical transformation of water molecules into active catalysts via protonation of water to form hydronium ions (allowing acid catalysis) and deprotonation to form hydroxide ions (allowing base catalysis; Supporting Fig. S1c) (Saá and Frontera 2020). Production of water in chemical reactions is common in chemical linkage and dehydration reactions; examples include linkages between and among acids, alcohols, amines, aldehydes or ketones, to form esters, peptides, or acetals (Supporting Fig. S1g). Changes in metal coordination chemically take up or release water. For example, when the ribosome assembles, rRNA coordinates Mg^2+^ ions and releases water from the first hydration shell of the metal (Bowman et al. 2012) (Supporting Fig. S1f). Release of water from divalent metal coordination is a form of chemical transformation (Capaldi and Aggeler 2002).

An example of the extensive involvement of water transformations in biochemistry is demonstrated in Fig. 1, which is a water-centric representation of the citric acid cycle (also termed the Krebs cycle). Except for the conversion of succinyl-CoA to succinate, water molecules chemically participate in each step of the citric acid cycle.

Of 172 metabolites listed in the Microbial Metabolome Database (Wishart et al. 2018), 163 contain at least 1 functional group capable of condensation dehydration and/or hydrolysis reactions. The central metabolite coenzyme A illustrates how deeply and completely the chemistry of water is integrated into metabolic processes. Coenzyme A can be hydrolyzed to form adenine, ribose, phosphate, pyrophosphate, β-alanine, β-mercaptoethylamine, and pantoic acid. Several hydroxyl groups, a thiol, and a phosphate of coenzyme A are available for condensation dehydration reactions.

### The Enzyme Commission Database

**Fig. 2:**
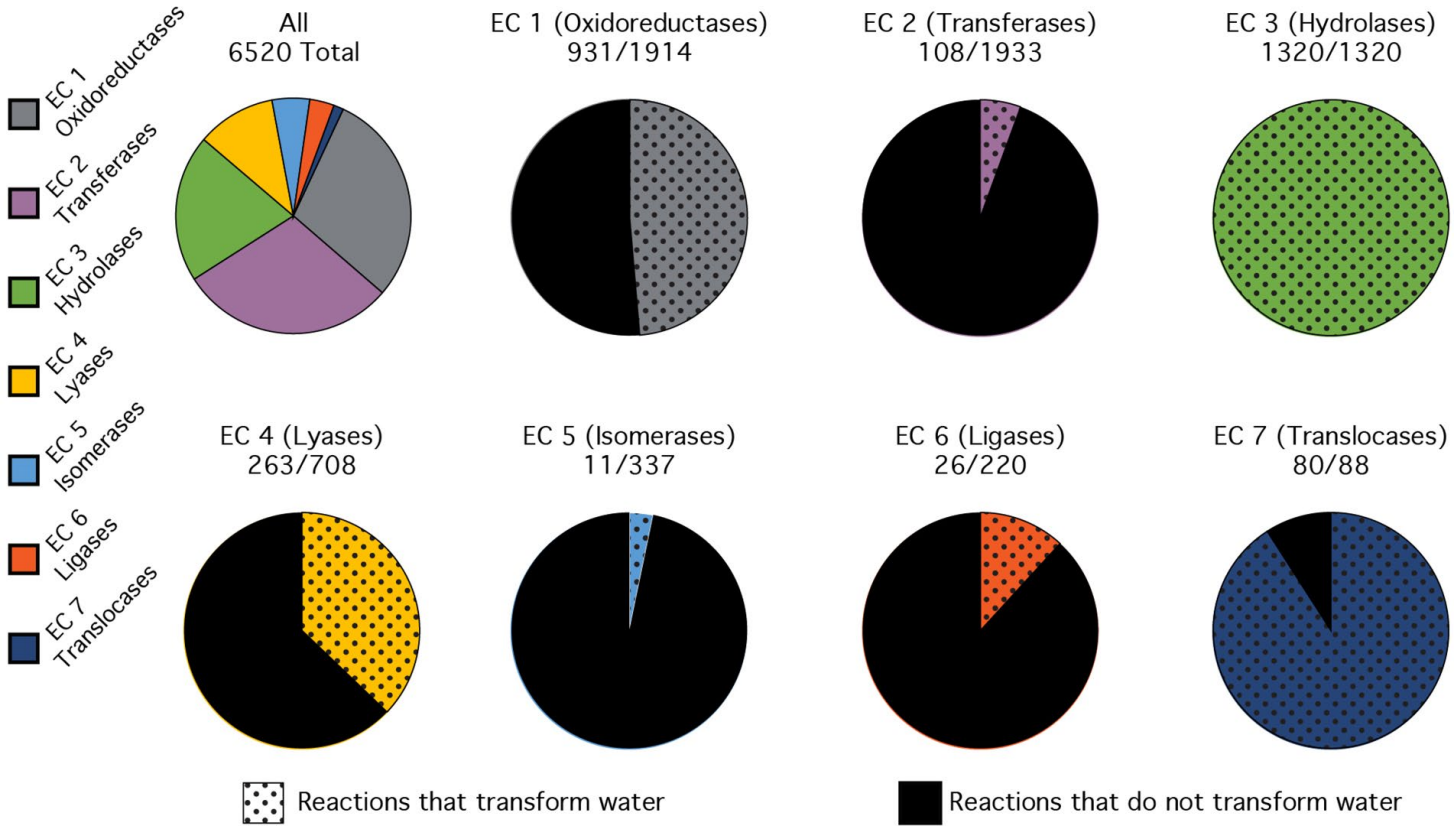
Many biological reactions involve transformations of water. A total of 42% (2739/6520) of Enzyme Commission (EC) reactions use water as either a substrate or a product. The distribution of reactions among seven EC classes is shown, along with the portion of water-transforming reactions in each class

The Enzyme Commission (Apweiler et al. 2010) (EC) classifies biochemical reactions by products and reactants, independent of enzyme mechanism or structure. A survey here indicated that between a third and a half (2739 out of 6520) of EC reactions contain water as either a substrate or a product (Fig. 2; Table S1). By contrast, the EC database contains fewer than 1000 reactions that consume or produce ATP and fewer than 460 reactions that consume or produce NAD^+^. The importance of water chemistry is also seen in alternative enzyme reaction classifications, such as those that classify reactions based on reactive site structures and catalytic mechanism. For example, approximately a half (445/879) of enzymatic reactions in the EzCatDB database (Nagano et al. 2015) consume or produce water.

### Quantitating Transformations of Water in *E. coli*

We calculated a lower limit for frequency of participation of water molecules in chemical transformation during replication of a single *E. coli*. We focused on protein production and oxidative phosphorylation with the assumption of oxic conditions in minimal medium. The estimate of the quantity of water transformed is a lower limit because it does not account for many water molecules used mechanistically during catalysis, those released or taken up by changes in metal coordination, those used in downstream chemical reactions of CO_2_ or H_2_O_2_, nor those chemically transformed outside of protein synthesis and oxidative phosphorylation. The focus was on protein synthesis and oxidative phosphorylation because they are dominant metabolic hubs (Russell and Cook 1995; Buttgereit and Brand 1995), requiring immense chemical energy and participation of many metabolites. Protein comprises approximately half of the dry weight of an *E. coli* (Milo 2013; Milo and Phillips 2015) and protein synthesis is a major metabolic consumer in biological systems (Stouthamer 1973). Biochemical reaction pathways and reaction mechanisms were inspected as outlined in detail in the “Materials and Methods” section. We tallied the number of water molecules that chemically participate in each step of these reaction pathways and counted the number of water molecules consumed or produced. Wherever possible, we identified water molecules involved mechanistically in catalytic function or as intermediates.

For protein synthesis, relevant reactions include those that produce amino acids from products of glycolysis and the citric acid cycle (Fig. 1; Supporting Figs. S3–S22), and those that polymerize amino acids to form proteins (see “Materials and Methods” section). Our results show that approximately 3.42 × 10^−14^ mol of water (6.16 × 10^−13^ g) participate in chemical transformations related to amino acid biosynthesis and amino acid polymerization as *E. coli* replicates. An *E. coli* cell contains about 3.9 × 10^−14^ mol of water. Therefore, about 88% of water molecules in an *E. coli* cell participate chemically in reactions of amino acid biosynthesis and polymerization.

*E. coli* living on glucose requires about 10 × 10^9^ ATP molecules (1.66 × 10^−14^ mol) to replicate (Farmer and Jones 1976; Phillips and Milo 2009). Many water molecules participate in the reactions of aerobic respiration, which produces about 1.45 × 10^−14^ mol of ATP during replication of an *E. coli* cell in the presence of O_2_ (Farmer and Jones 1976; Phillips and Milo 2009). Approximately, 28 out of 32 ATPs generated from one glucose molecule are produced through oxidative phosphorylation (Demirel 2013; Sokolov et al. 2015). As part of the oxidative phosphorylation pathway, ATP synthase catalyzes dehydration of ADP and Pi into an ATP molecule. In addition, ATP synthase uses two mechanistic water molecules for each proton that is translocated across the membrane from the periplasm to the cytoplasm (Capaldi and Aggeler 2002). Since 3.3 protons are required for the generation of 1 ATP molecule by ATP synthase (Capaldi and Aggeler 2002; Voet and Voet 2010; Hahn et al. 2016), and 2 water molecules are required mechanistically for the transport of each of those protons, a total of about 7 water molecules are involved in the generation of 1 ATP molecule by ATP synthase (including the dehydration of ADP and Pi into ATP). Moreover, during oxidative phosphorylation, there is delivery of electrons by the reduced NADH and FADH_2_ to O_2_. Dioxygen is split to form water. Taken together, we calculated that 1.1 × 10^−13^ mol (2.0 × 10^−12^ g) of water chemically participate in processes of oxidative phosphorylation during replication of an *E. coli* (see “Materials and Methods” section). The amount corresponds to 278% of the total number of water molecules in an *E. coli* cell, indicating that an average water molecule is repeatedly transformed.

Taken together, our results show that 1.4 × 10^−13^ mol of water chemically participate in protein synthesis and oxidative phosphorylation during the replication of an *E. coli*. This calculation implies that in the lower limit, an average water molecule is chemically transformed or is mechanistically involved in catalysis at least 3.7 times as an *E. coli* replicates. Our results demonstrate that an average biological water molecule is used repeatedly in chemical reactions during the life of a cell.

## Discussion

### Biochemistry is Chemistry of Water

**Fig. 3:**
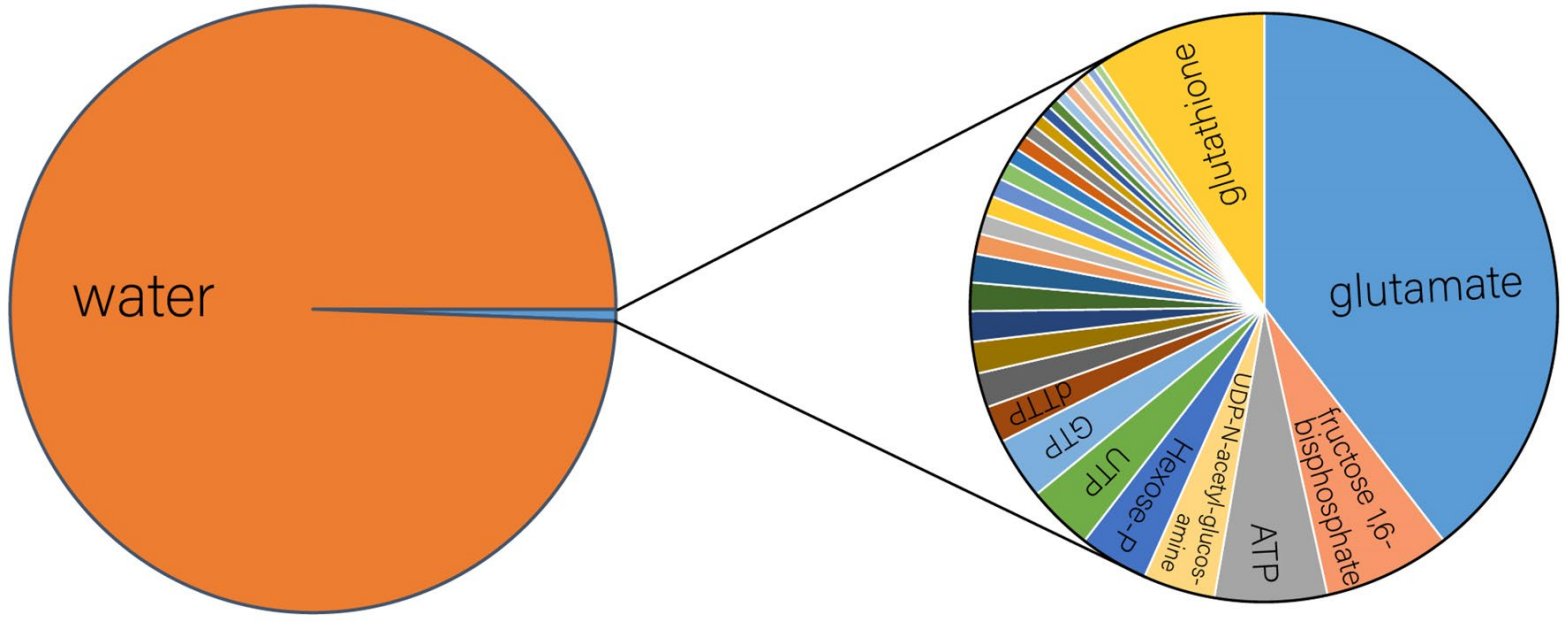
Water is a dominant metabolite in biochemistry, accounting for 99.4% by molarity of metabolites within an *E. coli*. Water in an *E. coli* cell is around 40 M (see “Materials and Methods” section). The sum of the concentrations of all other metabolites is 240 mM

The quantitative data show that water defines the chemistry of biology and is the dominant chemical actor in metabolism. Water accounts for 99.4% of metabolites in an *E. coli* by molar concentration (Fig. 3). The unifying theme of organic and inorganic molecules of biology is chemical production and/or consumption of water during their catabolism and anabolism. Polymer building blocks, polymers, and metabolites are chemically transformed by water. 42% of known enzymatic reactions chemically transform water (Fig. 2). For replication of an *E. coli* cell, the integrated flux of water through chemical processes appears to be greater for water than for any other molecule. Water is the reactive nexus of biology and the primary agent of chemical, thermodynamic, and kinetic linkage of biochemical processes.

A model of water as the reactive nexus of biology is qualitatively supported by the enormous number and diversity of enzymatic reaction types that produce or consume water and by the criticality of chemical transformations of water in reactions involving biopolymers, metals, and organic cofactors. The number of reaction types that transform water is far greater than the number of reaction types that transform other metabolites (such as ATP and NAD^+^). Chemical transformations of water are widespread in the core chemistry of life, including biosynthesis of amino acids, nucleotides, and membrane components (Voet and Voet 2010). Water is chemically transformed during information processing, as in the synthesis of DNA (Patel et al. 2011) and RNA (Belogurov and Artsimovitch 2015), and in synthesis of carbohydrates and phospholipids (Runnels et al. 2018). Transformations of water are also key to central metabolism, in glycolysis, the citric acid cycle, oxidative phosphorylation, and photosynthesis (Voet and Voet 2010).

A model of water as the reactive nexus of biology is quantitatively supported by the frequency of chemical transformations of water during replication of a single *E. coli* cell. The average water molecule participates repeatedly in these chemical transformations. Our quantification is a lower limit; we have not included waters used in processes such as synthesis of RNA, DNA, and membrane lipids. We did not count water molecules involved in changes in metal coordination. Moreover, mechanisms for many enzymes are not fully understood and might involve water molecules that were not accounted for here. Anaerobiosis is an important consideration; facultative anaerobes such as *E. coli* are capable of growth under both oxic and anoxic conditions (Tran and Unden 1998). Our quantitative results assume oxic conditions, whereas under anoxic conditions, anaerobic metabolic systems would transform water molecules in a pathway-dependent manner, to an extent that depends on availability of electron acceptors (such as nitrate and fumarate). Specifically, under anoxic conditions, if electron acceptors are available, *E. coli* would perform anaerobic respiration instead of fermentation; the former is expected to use a similar number of water molecules as aerobic respiration, mostly due to the heavily water-consuming, ATP synthase, which is common to both types of respiration pathways (Ingledew and Poole 1984). On the other hand, if fermentation is used as the major energy-producing pathway, fewer transformations of water molecules would occur because fermentation does not involve ATP synthesis through ATPase.

### General Implications for Biology

Water is (i) a unique medium that promotes complex molecular assemblies (Ball 2017), (ii) highly reactive (Brack 1993), (iii) the dominant metabolite, and (iv) an agent in chemical transformations of a great majority of biological molecules. Water forms a continuum from medium to chemical participant. By extent of chemical participation, water dominates the chemistry of biology at a fundamental level. The sharing of water molecules as a common substrate, intermediate, and/or product provides mechanisms of chemical, thermodynamic, and kinetic linkage across an enormous number of reactions. Incorporation of water as a metabolite into existing metabolite maps (such as the IUBMB-Nicholson Metabolic Maps) would reveal a dense network of associations. Broad analysis of water metabolism across various pathological conditions has not been established thus far and might reveal interesting water-related metabolic defects that could contribute to disease development.

A large number of water molecules are observed in X-ray structures, linking enzymes with substrates, intermediates, effectors or products. These water molecules beautifully complement enzyme–ligand complexes that encompass them. For example, many water molecules form specific hydrogen bonds that mediate interactions between nucleotide substrates and succinyl coenzyme A synthetase, an enzyme in the citric acid cycle that interconverts succinyl-CoA and succinate. Are these waters part of the medium or are they specific actors in a reaction mechanism? They are both, there is no distinction.

### The Origins of Life on Earth

The centrality of water in biochemistry, both as a medium and in chemical processes, can help us understand prebiotic chemistry and the chemical origins of life*.* A simple explanation for what one observes in extant biochemistry is that prebiotic chemical selection, leading to life, was substantially directed by water. Water was a primary gate-keeper that allowed in or denied access to organic molecules and metals during prebiotic chemistry. Specifically, chemical evolution selected building blocks that (i) were soluble in water, (ii) were chemically transformed by water, (iii) chemically transformed water, (iv) joined covalently, by condensation–dehydration reactions with near-equilibrium thermodynamics with accessible kinetics. Building blocks that participated in water chemistry and were chemically linked via water chemistry were selected to form small chimeras (e.g., triglycerides, coenzyme A, or nucleotides) and large homogeneous polymers (DNA, RNA, protein, polysaccharide). Potential building blocks that did not participate in water chemistry were excluded. This scenario explains the exclusion of various molecules from extant biochemistry, such as polycyclic aromatic hydrocarbons, which are highly abundant in the abiotic universe (Hertzog et al. 2019; Li 2020), but do not react with water.

### Proto-Metabolism

The activity of water on land surfaces of our rotating planet is in fluctuating disequilibrium, causing oscillations in directionalities of water-based chemical reactions. These geochemical phenomena are replicated by experimental “wet-dry chemistry” (Mamajanov et al. 2014; Forsythe et al. 2015; Ross and Deamer 2016; Frenkel-Pinter et al. 2019; Doran et al. 2019), which uses chemical transformations of water to build proto-biological oligomers. In these experiments, environmental energy is transduced to chemical energy by near-equilibrium, kinetically accessible reactions. Thus wet–dry chemistry can be considered a form of water-centric proto-metabolism.

Near-equilibrium, oscillating reactions driven by cycling water activity provide experimental platforms for chemical evolution, allowing the exploration of open-ended chemical change. Oligomers form and degrade; those that fold and assemble degrade more slowly, increase in relative population (Runnels et al. 2018). These experimental systems link proto-metabolism to biopolymer synthesis. It seems likely that metabolism co-evolved with polymers, neither was first.

### Summary

The Turkish novelist Mehmet Murat ildan said, “Water is the most perfect traveler because when it travels it becomes the path itself.” American poet Lucy Larcom said, “A drop of water, if it could write out its own history, would explain the universe to us.” Water is transformative and is transformed, eternally causing change and constantly changing. Water is the gate-keeper and the matrix. This duality is seen from planets, during formation of oceans and tectonic plates, to cells, during energy transduction and biopolymer assembly. We suggest that intense chemical participation of the medium might be an important characteristic of living systems in general. It is possible that other liquids, such as ammonia at low temperatures, or organic eutectics, might meet these requirements.

The picture that emerges from this analysis is of enzymatic systems integrated with each other by chemical transformations of the medium. The unusual physical properties of water as a solvent are coupled with frequent participation in diverse chemical transformations during the life of a cell. Water is never absent or physically separated from biological macromolecules, organic cofactors, and metals, but readily combines with, withdraws from, and intercedes in their transformations. In biological systems, water is fully integrated into processes of bond making and bond breaking. The distinction between medium and chemical participant is so blurred as to be devoid of meaning. This quantitative demonstration of water as the dominant metabolite in biochemistry suggests, based on expectations of continuity in evolution, that origins of life studies should focus on scenarios where the chemistry of water is central to emergence of biopolymers, organic metabolites, and metabolism. Leonardo da Vinci was correct: water is the ultimate vehicle of nature.

## Materials and Methods

### General Approach to Analysis of Water biochemistry

To calculate the number of water molecules that participate in chemical transformation during replication of a single *E. coli* (a doubling event), we focused on protein synthesis and oxidative phosphorylation. These two processes involve the greatest pluralities of chemical transformations within a cell and present the greatest energy flux. We assume that replication of an *E. coli* requires reproduction of every chemical species in an *E. coli*, except those that are supplied by the media. We analyzed metabolic processes required to make these chemical species. Our results here are for an *E. coli* that is replicated on minimal media under aerobic condition. We presume standard M9 minimal media (Anonymous 2010). Changes in media and in oxygen availability will change the specific numbers here, but will not change the general conclusion that water is intimately involved in chemical processes in living systems and is a predominant metabolite in biochemistry.

The water molecules accounted for here, as chemical participants, are either directly consumed or produced in transformation of substrate into product, or are involved mechanistically in the catalytic activity of the enzymes as intermediates or as catalysts. The role of water molecules in enzyme mechanism is commonly uncharacterized. Therefore, the numbers of catalytic waters should be considered as underestimates. = Water has been proposed as a general catalyst in prebiotic chemistry (Saá and Frontera 2020).

Our attempt to quantify the number of water molecules involved in cellular metabolism is inspired in part by the general approach taken in Cell Biology by the Numbers (Milo and Phillips 2015).

### Transformations of Water During Protein Synthesis

Protein constitutes about 52% of the dry weight of an *E. coli* (Milo 2013; Milo and Phillips 2015). We calculated the number of water molecules that are chemically involved in the biosynthesis of each of the 20 amino acids in an *E. coli* and weighted the results by relative amino acid abundance in *E. coli.* In addition, we determined the number of water molecules that chemically participate in polymerization of amino acids into protein.

Our calculations took the frequency of each amino acid in the *E. coli* K-12 proteome into account, based on Proteome-pI database:

**Table:** 

Amino acid	Frequency (%)
Ala	9.52
Cys	1.16
Asp	5.15
Glu	5.76
Phe	3.89
Gly	7.37
His	2.27
Ile	6.01
Lys	4.41
Leu	10.67
Met	2.82
Asn	3.95
Gln	4.44
Pro	4.43
Arg	5.51
Ser	5.8
Thr	5.4
Val	7.07
Trp	1.53
Tyr	2.85

*Source* Kozlowski (2017)

The total number of amino acids in *E. coli* used here are obtained from Milo (2013) and Milo and Phillips (2015).

**Table:** 

Number of proteins in an *E. coli*	3.50 × 10^6^
Average number of AA/protein	3.00 × 10^2^
Total number of AAs in an *E. coli*	1.05 × 10^9^

Based on the relative frequency of the amino acids (AAs) in the *E. coli* proteome, the total number of proteinogenic amino acids incorporated into protein (1.05 × 10^9^), the number of water molecules that are involved in synthesis of each of the 20 amino acids (Supporting Figs. S3, S4, S5, S6, S7, S8, S9, S10, S11, S12, S13, S14, S15, S16, S17, S18, S19, S20, S21, S22), and the number of water molecules per *E. coli* (2.34 × 10^10^), we calculated the percentage of water molecules that are involved in amino acid synthesis pathways.

Total number of an AA in protein = [frequency of the AA in protein × total number of AAs incorporated into protein in an *E. coli* (1.05 × 10^9^)].

Total number of water molecules involved in the synthesis of an AA = total number of an AA in protein × number of water molecules involved in the synthesis of that AA.

Percent of total water molecules in an *E. coli* involved in AA synthesis = (total number of water molecules involved in the synthesis of an AA × 100)/total number of water molecules involved in the synthesis of the AA by the total number of cellular water molecules (2.34 × 10^10^).

The metabolic pathways for amino acid biosynthesis depicted here were based on the KEGG database for *E. coli* K-12 MG1655 strain (Kanehisa et al. 2007).

**Table:** 

Amino acid	Frequency of AA in proteome (%)	Total # of AAs	Water molecules/AA	Total # of water molecules	Total # of water as percent of water in *E. coli* (%)
Ala	9.52	1.00 × 10^8^	6.5	6.50 × 10^8^	2.78
Cys	1.16	1.22 × 10^7^	7.5	9.15 × 10^7^	0.39
Asp	5.15	5.41 × 10^7^	19.5	1.05 × 10^9^	4.51
Glu	5.76	6.05 × 10^7^	12.5	7.56 × 10^8^	3.23
Phe	3.89	4.08 × 10^7^	15.5	6.32 × 10^8^	2.70
Gly	7.37	7.74 × 10^7^	10.5	8.13 × 10^8^	3.47
His	2.27	2.38 × 10^7^	15	3.57 × 10^8^	1.53
Ile	6.01	6.31 × 10^7^	34	2.15 × 10^9^	9.17
Lys	4.41	4.63 × 10^7^	36	1.67 × 10^9^	7.12
Leu	10.67	1.12 × 10^8^	16	1.79 × 10^9^	7.66
Met	2.82	2.96 × 10^7^	26.5	7.84 × 10^8^	3.35
Asn	3.95	4.15 × 10^7^	21.5	8.92 × 10^8^	3.81
Pro	4.43	4.65 × 10^7^	15.5	7.21 × 10^8^	3.08
Gln	4.44	4.66 × 10^7^	12.5	5.83 × 10^8^	2.49
Arg	5.51	5.79 × 10^7^	19.5	1.13 × 10^9^	4.83
Ser	5.8	6.09 × 10^7^	6.5	3.96 × 10^8^	1.69
Thr	5.4	5.67 × 10^7^	23.5	1.33 × 10^9^	5.69
Val	7.07	7.42 × 10^7^	12	8.90 × 10^8^	3.81
Trp	1.53	1.61 × 10^7^	18.5	2.98 × 10^8^	1.27
Tyr	2.85	2.99 × 10^7^	14.5	4.34 × 10^8^	1.85
Sum		1.05 × 10^9^		1.74 × 10^10^	74.4

Polymerization of amino acids into protein by the ribosome involves three water molecules per amino acid (Voet and Voet 2010): two of which are involved in GTP hydrolysis and one comes from hydrolysis of PPi following the amino acid adenylate formation.

In one *E. coli* there are 1.05 × 10^9^ amide bonds (Milo 2013; Milo and Phillips 2015). The number of water molecules used for amino acid polymerization = 3 (water molecules per polymerization of a single amino acid into protein) × 1.05 × 10^9^ (total number of amide bonds in proteins in an *E. coli*) = 3.15 × 10^9^ water molecules. An *E. coli* contains 2.34 × 10^10^ water molecules (Milo and Phillips 2015), which is 3.89 × 10^−14^ mol.

For amino acid polymerization an *E. coli* uses 3.15 × 10^9^ water molecules, which amounts to 13.5% of the total number of water molecules within the *E. coli* [(3.15 × 10^9^/2.34 × 10^10^) × 100]. To sum up the amount of water molecules for synthesis of the amino acids and their subsequent polymerization, we add up the following processes. The % of *E. coli* water molecules used for protein synthesis = % water molecules used for amino acid synthesis + % water molecules for polymerization of amino acids = 74.4% + 13.5% = 87.9%. If the doubling time of *E. coli* is 20 min, then the average water molecule in *E. coli* is chemically transformed during protein synthesis 0.879/0.33 = 2.66 times per hour. *E. coli* has 2.34 × 10^10^ water molecules (Milo and Phillips 2015), which is 3.89 × 10^−14^ mol. 3.42 × 10^−14^ mol of water are utilized in protein synthesis (= 6.16 × 10^−13^ g). Given a cell volume of 1 × 10^−15^ (4), water molarity in an *E. coli* is 38.9 M.

### Water Transformations During Oxidative Phosphorylation

*Escherichia coli* growing on glucose requires about 10 × 10^9^ ATP molecules to replicate (Farmer and Jones 1976; Phillips and Milo 2009), which is 1.66 × 10^−14^ mol of ATP. Under oxic conditions, *E. coli* invests in aerobic respiration over fermentation and anaerobic respiration. That is, 28 out of 32 molecules that are generated from a single glucose will be produced through oxidative phosphorylation (= 8.75 × 10^9^ ATP molecules) (Demirel 2013; Sokolov et al. 2015). The other 4 ATP molecules are generated through substrate-level phosphorylation.

As part of the oxidative phosphorylation pathway, the ATP synthase catalyzes dehydration of ADP and Pi into one ATP molecule (one water molecule is released during this process). In addition, ATP synthase has two mechanistic water molecules that are involved in each proton translocation across the membrane from the periplasm to the cytoplasm (Ribeiro et al. 2018). Since ~ 3.3 protons are required for the generation of one ATP molecule by the ATP synthase (Voet and Voet 2010), and two water molecules are required mechanistically for the transport of each of those protons, then a total of about seven water molecules are required for the generation of one ATP molecule by the synthase. Given that 8.75 × 10^9^ ATP molecules result from oxidative phosphorylation and that 7 water molecules per one ATP molecule are utilized by the ATP synthetase, then 6.125 × 10^10^ water molecules are involved in oxidative phosphorylation in *E. coli*. Since *E. coli* has 2.34 × 10^10^ water molecules, ~ 262% of *E. coli*’s water molecules are involved in this process of ATP production through aerobic respiration by the ATP synthase.

In addition, during oxidative phosphorylation there is delivery of electrons by the reduced NADH and FADH_2_ molecules to O_2_. The oxygen is being split to form water. Approximately 2 NADH molecules result in the formation of 5 molecules of ATP and 2 water molecules (2 NADH = 2 H_2_O = 5 ATP → ATP = 0.4 H_2_O from NADH) (51). Approximately 2 molecules of FADH_2_ result in the formation of 3 ATP molecules and 2 water molecules (2 FADH_2_ = 2 H_2_O = 3 ATP → ATP = 0.66 H_2_O from FADH_2_) (Manoj et al. 2019). Out of the 28 ATP molecules that are generated per 1 glucose molecule through oxidative phosphorylation, approximately 26 ATP molecules will be generated through NADH, and 2 ATP molecules will be generated through FADH_2_. As 8.75 × 10^9^ ATP molecules result from oxidative phosphorylation in *E. coli*, 8.125 × 10^9^ [= (26/28) × 8.75 × 10^9^)] are generated through NADH and 0.625 × 10^9^ [= (2/28) × 8.75 × 10^9^)] are generated through FADH_2_. Such being the case, generation of ATP molecules through NADH will result in the transformation of 8.125 × 10^9^ × 0.4 water molecules (= 3.25 × 10^9^), whereas generation of ATP molecules through FADH_2_ will result in the transformation of 0.625 × 10^9^ × 0.66 water molecules (= 0.42 × 10^9^). Summed up together, generation of ATP molecules though splitting of oxygen mediated by NADH and FADH_2_ results in the formation of 3.67 × 10^9^ water molecules. Since *E. coli* has 2.34 × 10^10^ water molecules, 15.7% of *E. coli* water molecules are generated via splitting of oxygen.

To summarize, 278% of the water molecules of an *E. coli* are chemically transformed during oxidative phosphorylation. That is 6.50 × 10^10^ molecules of water, or 1.08 × 10^−13^ mol, or (= 1.95 × 10^−12^ g). If the doubling time of *E. coli* is 20 min, then the average water molecule in *E. coli* is chemically transformed during oxidative phosphorylation 2.78/0.33 = 8.3 times/h.

## Electronic supplementary material

Below is the link to the electronic supplementary material.

(DOCX 8084 kb)
