# Supplementary material for: Water and Life: The Medium is the Message
Source: J Mol Evol. 2021 Jan 11;89(1):2–11. doi: 10.1007/s00239-020-09978-6 (PMC7884305; doi:10.1007/s00239-020-09978-6)
Supplement: Supplementary file 1 — (DOCX 8084 kb) [file 239_2020_9978_MOESM1_ESM.docx]

**Supporting Information**

**Water and Life: The Medium is the Message**

Moran Frenkel-Pinter^1,2,3^, Vahab Rajaei^1,2,3^, Jennifer B. Glass^1,4^, Nicholas V. Hud^1,2,3^, and Loren Dean Williams*^1,2,3^

^1^NASA Center for the Origins of Life, ^2^NSF-NASA Center of Chemical Evolution, ^3^School of Chemistry and Biochemistry, Georgia Institute of Technology, Atlanta, GA 30332-0400 USA, ^4^School of Earth and Atmospheric Science, Georgia Institute of Technology, Atlanta, GA 30332-0400 USA

* direct correspondence to this author.

Table of Contents

[Physical Properties of H_2_O. 4](#_Toc55932331)

[Physical Properties of D_2_O. 4](#_Toc55932332)

[Table S1. Enzyme Commission reactions that chemically consume or produce water. 5](#_Toc55932333)

[Supplementary Figures 6](#_Toc55932334)

[Fig. S1. Chemical transformations of water in biochemical reactions. 6](#_Toc55932335)

[Fig. S2. Water molecules form dense networks of near ideal hydrogen bonds. 7](#_Toc55932336)

[Fig. S3. Alanine biosynthesis chemically transforms 6.5 water molecules. 8](#_Toc55932337)

[Fig. S4. Cysteine biosynthesis chemically transforms 7.5 water molecules. 9](#_Toc55932338)

[Fig. S5. Aspartic acid biosynthesis chemically transforms 19.5 water molecules. 10](#_Toc55932339)

[Fig. S6. Glutamic acid biosynthesis chemically transforms 12.5 water molecules. 11](#_Toc55932340)

[Fig. S7. Phenylalanine biosynthesis chemically transforms 15.5 water molecules. 12](#_Toc55932341)

[Fig. S8. Glycine biosynthesis chemically transforms 10.5 water molecules. 13](#_Toc55932342)

[Fig. S9. Histidine biosynthesis chemically transforms 15 water molecules. 14](#_Toc55932343)

[Fig. S10. Isoleucine biosynthesis chemically transforms 34 water molecules. 15](#_Toc55932344)

[Fig. S11. Lysine biosynthesis chemically transforms 36 water molecules. 16](#_Toc55932345)

[Fig. S12. Leucine biosynthesis chemically transforms 16 water molecules. 17](#_Toc55932346)

[Fig. S13. Methionine biosynthesis chemically transforms 26.5 water molecules. 18](#_Toc55932347)

[Fig. S14. Asparagine biosynthesis chemically transforms 21.5 water molecules. 19](#_Toc55932348)

[Fig. S15. Proline biosynthesis chemically transforms 15.5 water molecules. 20](#_Toc55932349)

[Fig. S16. Glutamine biosynthesis chemically transforms 12.5 water molecules. 21](#_Toc55932350)

[Fig. S17. Arginine biosynthesis chemically transforms 19.5 water molecules. 22](#_Toc55932351)

[Fig. S18. Serine biosynthesis chemically transforms 6.5 water molecules. 23](#_Toc55932352)

[Fig. S19. Threonine biosynthesis chemically transforms 23.5 water molecules. 24](#_Toc55932353)

[Fig. S20. Valine biosynthesis chemically transforms 12 water molecules. 25](#_Toc55932354)

[Fig. S21. Tryptophan biosynthesis chemically transforms 18.5 water molecules. 26](#_Toc55932355)

[Fig. S22. Tyrosine biosynthesis chemically transforms 14.5 water molecules. 27](#_Toc55932356)

[SI References 28](#_Toc55932357)

# Physical Properties of H_2_O.

Water has unique physical properties that allow it to play a dual role as a medium and metabolite. Water has a high boiling point, a capacity to absorb heat with minimal temperature change, and a tendency to cause aggregation of non-polar substances (Edsall and McKenzie 1983; Eisenberg and Kauzmann 2005; Sharp and Vanderkooi 2010). Water is the most common substance seen on Earth as [solid](https://en.wikipedia.org/wiki/Ice), liquid, or [gas](https://en.wikipedia.org/wiki/Water_vapor), and solubilizes and dissociates many polar and charged species. The unusual properties of liquid water arise from directional cohesive hydrogen bonding (HB) interactions between water molecules (Figure S2). A water molecule is self-complementary and symmetrical, with two HB donors balanced by two HB acceptors. Liquid and solid water contain a dense network of near-ideal HBs; donors are geometrically matched with acceptors. A water molecule averages four ideal HBs with neighboring water molecules at 0°C in the liquid (Lee and Tuckerman 2007; Sharp and Vanderkooi 2010). The HB network of water is dynamic; molecules rotate, and covalent and hydrogen bonds interconvert. The lifetime of a hydrogen bond is around 10 picoseconds in liquid water at 0°C (Lee and Tuckerman 2007).

# Physical Properties of D_2_O.

The density of D_2_O is 1.1 times greater than that of water. The dissociation constant of D_2_O is smaller than of H_2_O, and the solubility of the organic and inorganic substances in D_2_O is less than in H_2_O (Kirshenbaum 1948). The strength of hydrogen-oxygen bonds is different between D_2_O and H_2_O. Differences in physical properties of H_2_O and D_2_O give rise to the “kinetic isotope effect”, a phenomenon which causes differences in reaction kinetics in D_2_O and H_2_O. For instance, rates of acid-catalyzed reactions are usually two to three times greater in D_2_O than in H_2_O (Laidler 1987).

# Table S1. Enzyme Commission reactions that chemically consume or produce water.

| EC Group | Reactions that consume or produce water |
| --- | --- |
| 1 | **Oxidoreductases (931/1914)** |
| 2 | **Transferases (108/1933)** |
| 3 | **Hydrolases (1320/1320)** |
| 3.1 | cleavage of esters |
| 3.2 | cleavage of polysaccharides/nucleosides |
| 3.3 | cleavage of ethers |
| 3.4 | cleavage of peptides |
| 3.5 | cleavage of carbon-nitrogen bonds |
| 3.6 | cleavage of acid anhydrides |
| 3.7 | cleavage of carbon-carbon bonds |
| 3.8 | cleavage of halide bonds |
| 3.9 | cleavage of phosphorous-nitrogen bonds |
| 3.10 | cleavage of sulfur-nitrogen bonds |
| 3.11 | cleavage of carbon-phosphorous bonds |
| 3.12 | cleavage of sulfur-sulfur bonds |
| 3.13 | cleavage of carbon-sulfur bonds |
| 4 | **Lyases (263/708)** |
| 5 | **Isomerases (11/337)** |
| 6 | **Ligases (26/220)** |
| 7 | **Translocases (80/88)** |

# Supplementary Figures


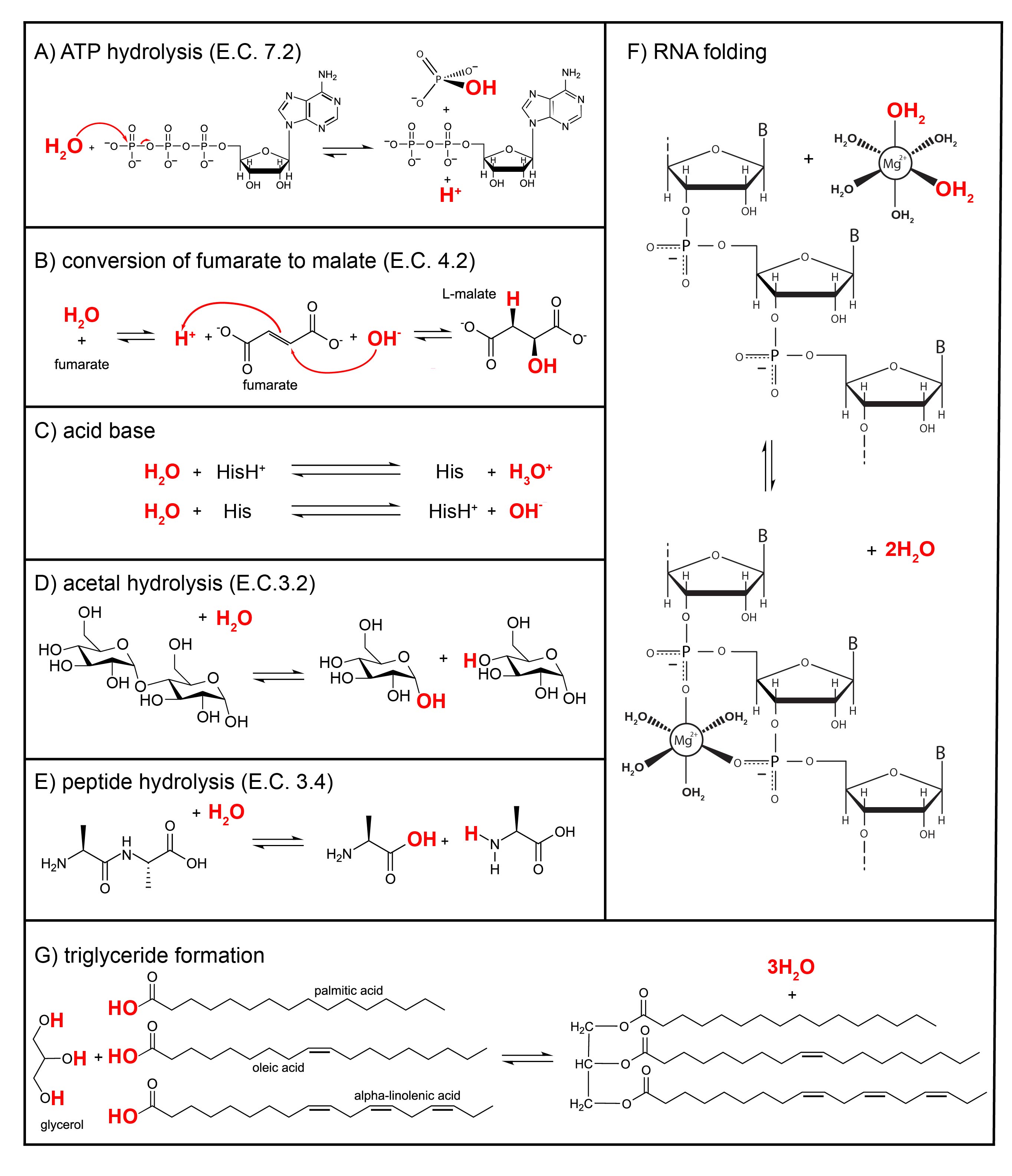


**Fig. S1.** Chemical transformations of water in biochemical reactions. (A) Addition of a water molecule causes hydrolysis of ATP to form ADP and Pi; (B) in the citric acid cycle (TCA cycle) fumarate is hydrolyzed to L-malate; (C) water molecules are protonated by histidine to form hydronium ions and deprotonated by histidine to form hydroxide ions; (D) cleavage of maltose by addition of a water molecule to from two glucose molecules; (E) a dipeptide is cleaved by the addition of a water molecule to form two amino acids; (F) assembly of the ribosome partially dehydrates magnesium ions by chelation with backbone phosphate groups, releasing water from the first coordination sphere; and (G) glycerol and three carboxylic acids (palmitic acid, oleic acid and alpha-linolenic acid) are condensed to form triglyceride, the main constituent of body fat, producing three water molecules.


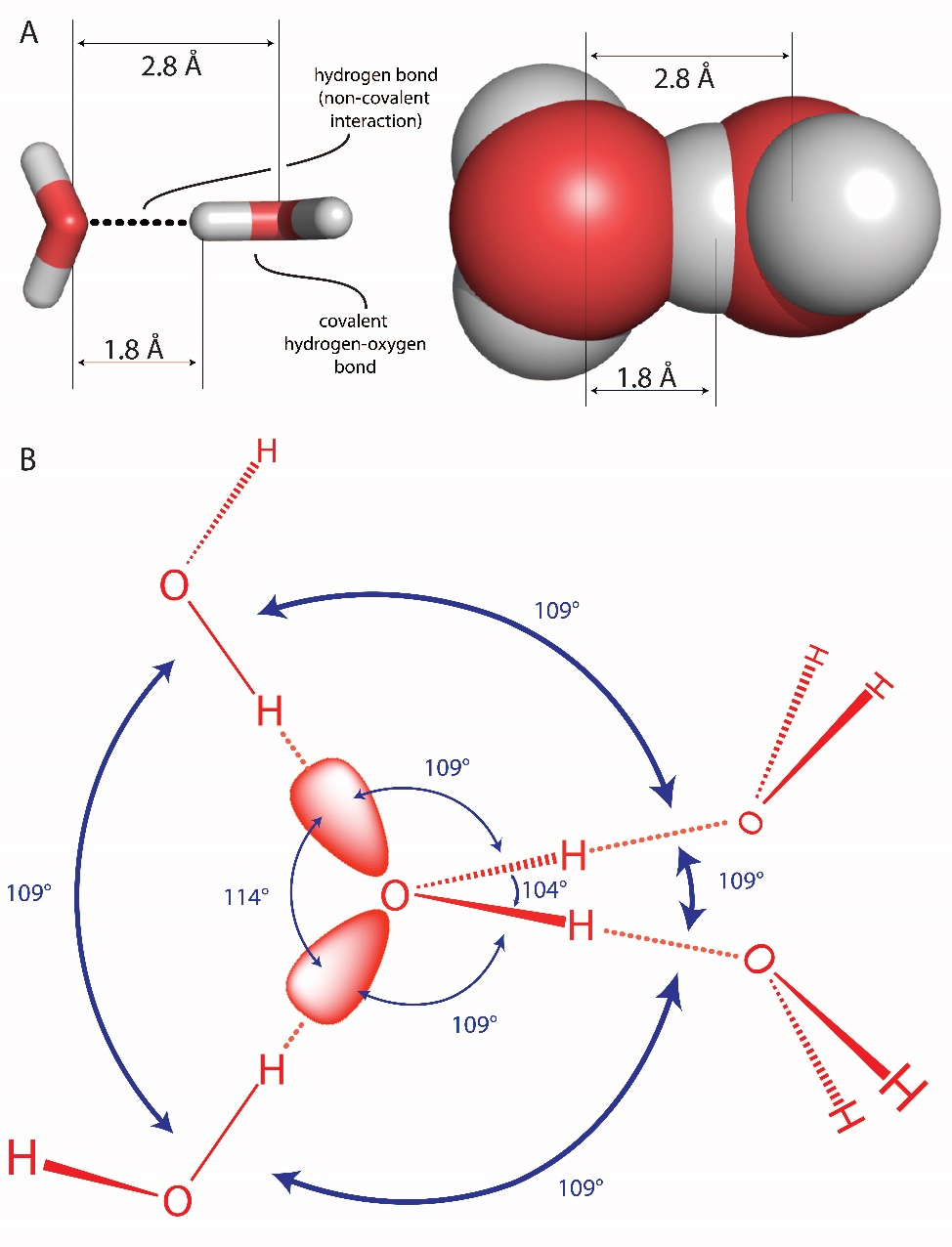


**Fig. S2.** Water molecules form dense networks of near ideal hydrogen bonds. (A) Illustration of the difference between a covalent oxygen-hydrogen bond and a hydrogen bond, which is a non-covalent interaction. Oxygen atoms are red and hydrogen atoms are white. The space filling representation on the right shows how hydrogen bonding causes violations of van der Waals surfaces. The ideal hydrogen-bonding distance from H to O is around 1.8 Å, which is less than the sum of the O and H van der Waals radii (rO=1.5 Å; rH=1.0 Å). (B) A water molecule is balanced, with two HB donor sites and two HB acceptor sites. The hydrogen bond functionalities of a water molecule are complementary with those of the surrounding water molecules in the liquid and solid.

## **Fig. S3.** Alanine biosynthesis chemically transforms 6.5 water molecules.

## **Fig. S4.** Cysteine biosynthesis chemically transforms 7.5 water molecules.

## **Fig. S5.** Aspartic acid biosynthesis chemically transforms 19.5 water molecules.

## **Fig. S6.** Glutamic acid biosynthesis chemically transforms 12.5 water molecules.

## **Fig. S7.** Phenylalanine biosynthesis chemically transforms 15.5 water molecules.

## **Fig. S8.** Glycine biosynthesis chemically transforms 10.5 water molecules.

## **Fig. S9.** Histidine biosynthesis chemically transforms 15 water molecules.

## **Fig. S10.** Isoleucine biosynthesis chemically transforms 34 water molecules.

## **Fig. S11.** Lysine biosynthesis chemically transforms 36 water molecules.

## **Fig. S12.** Leucine biosynthesis chemically transforms 16 water molecules.

## **Fig. S13.** Methionine biosynthesis chemically transforms 26.5 water molecules.

## **Fig. S14.** Asparagine biosynthesis chemically transforms 21.5 water molecules.

## **Fig. S15.** Proline biosynthesis chemically transforms 15.5 water molecules.

## **Fig. S16.** Glutamine biosynthesis chemically transforms 12.5 water molecules.

## **Fig. S17.** Arginine biosynthesis chemically transforms 19.5 water molecules.

## **Fig. S18.** Serine biosynthesis chemically transforms 6.5 water molecules.

## **Fig. S19.** Threonine biosynthesis chemically transforms 23.5 water molecules.

## **Fig. S20.** Valine biosynthesis chemically transforms 12 water molecules.

## **Fig. S21.** Tryptophan biosynthesis chemically transforms 18.5 water molecules.

## **Fig. S22.** Tyrosine biosynthesis chemically transforms 14.5 water molecules.

# SI References

Edsall J, McKenzie H (1983) Water and proteins. II. The location and dynamics of water in protein systems and its relation to their stability and properties. Advances in biophysics 16:53-183.

Eisenberg D, Kauzmann W (2005) The structure and properties of water. Oxford University Press, Oxford.

Kirshenbaum I (1948) Physical properties of heavy water. United States Atomic Energy Commission, Technical Information Division

Laidler KJ (1987) Chemical kinetics. Harper & Row 3rd Edition

Lee H-S, Tuckerman ME (2007) Dynamical properties of liquid water from ab initio molecular dynamics performed in the complete basis set limit. The Journal of chemical physics 126:164501.

Sharp KA, Vanderkooi JM (2010) Water in the half shell: Structure of water, focusing on angular structure and solvation. Acc. Chem. Res. 43:231-239.
